# Evaluation of patients’ expectations and benefits in the treatment of allergic rhinitis with a new tool: the patient benefit index – the benefica study

**DOI:** 10.1186/s13223-015-0073-1

**Published:** 2015-02-26

**Authors:** Pascal Demoly, Michel Aubier, Frédéric de Blay, François Wessel, Pierre Clerson, Pascal Maigret

**Affiliations:** Department of Pulmonology - Division of Allergy, Hôpital Arnaud de Villeneuve, University Hospital of Montpellier, 34295 Montpellier cedex 5, France and Sorbonne Universités, UPMC Paris 06, UMR-S 1136, IPLESP, Equipe EPAR, 75013 Paris, France; Service de Pneumologie, Hôpital Bichat, Assistance Publique - Hôpitaux de Paris, Paris, France; Université Paris Diderot - Paris 7, Paris, France; INSERM Unité 700, Faculté de Médecine Bichat, Paris, France; Department of Chest Diseases, University Hospital of Strasbourg, Strasbourg, France; L’institut du thorax - INSERM U 915, Nantes, France; Orgamétrie Biostatistiques, Roubaix, France; Medical Department, Menarini, Rungis, France

**Keywords:** Allergic rhinitis, Satisfaction, Patients’ needs, Treatment-related benefit, Antihistamine, AR-PBI, Patient benefit index, Real-life study

## Abstract

**Background:**

Symptoms of allergic rhinitis (AR) have a detrimental effect on quality of life. The AR-Patient Benefit Index (AR-PBI), a specific self-assessment tool has been developed to assess treatment-related benefit in two separate sections: the Patient Needs Questionnaire (PNQ) which explores the patient’s expectations before treatment and the Patient Benefit Questionnaire (PBQ) which evaluates treatment benefit. For the PNQ, three dimensions summarize patients’ expectations: symptoms, social life and emotional state, thus covering a larger field than symptomatic relief. The aim of the study was to validate the French language version of the AR-PBI and to assess the treatment-related expectations and benefits provided in patients with allergic rhinitis treated with H1-antihistamines in a real-life study.

**Methods:**

BENEFICA was a prospective, observational study involving patients with allergic rhinitis who were starting treatment with H1-antihistamines. The Patient Needs Questionnaire (PNQ) was administered before treatment (D0) and the Patient Benefit Questionnaire (PBQ) was collected after a 14-day course of H1-antihistamines (D15). Discomfort (visual analog scale), and quality of life (miniRQLQ) were measured on D0 and D15.

**Results:**

Three thousands and eighty-nine patients were enrolled in the study: mean age 39 ± 14 years, women 52%, 81% of patients with moderate to severe persistent rhinitis (Allergic Rhinitis and its Impact on Asthma, ARIA); 19% had (a) concomitant condition(s), 18% were asthmatic, and 12% had atopic dermatitis. Discomfort and quality of life improved between D0 and D15. AR-PBI was 2.7 ± 0.8, superior to 1 (threshold for clinically relevant benefit) for 97% of patients and greater in patients willing to continue the treatment. PBI was moderately correlated to change in miniRQLQ (r = −0.45, p < 0.0001) and change in discomfort (r = −0.38, p < 0.0001), suggesting a richer conceptual content than symptoms relief.

**Conclusions:**

The French version of the Allergic Rhinitis-Patient Benefit Index (AR-PBI) has been validated. It complements the discomfort and quality of life tools and assesses the needs and benefits in patients suffering from allergic rhinitis. This new tool may help physicians to better understand patients’ expectations and to discuss treatment issues with their patients.

**Electronic supplementary material:**

The online version of this article (doi:10.1186/s13223-015-0073-1) contains supplementary material, which is available to authorized users.

## Background

Allergic rhinitis (AR) is one of the most common manifestations of immunoglobulin E (IgE)-mediated inflammation after allergen exposure of the nasal mucosa membrane. It is clinically defined as a symptomatic disorder of the nose characterized by the association of rhinorrhea, sneezing, nose stinging, and nasal congestion, frequently associated with symptoms such as conjunctival/pharyngeal stinging and eye redness.

AR is very common in Western countries but it frequently remains underdiagnosed [1]. In France, prevalence was found to be 31% in adults in a well-conducted study involving a representative random sample of more than 10,000 subjects [2]. Prevalence is higher in young people and its frequency decreases with age [3].

AR impairs quality of life, sleep and social activities [2,4]. It is a significant cause of reduced work productivity and lost school days. Poor sleep quality may induce diurnal somnolence. Impact is correlated with the severity of symptoms. AR is frequently associated with several co-morbidities, including asthma, and physicians are encouraged to ask AR patients about symptoms of asthma [5].

Several medications are available for AR and they are often prescribed in combination [6]. Antihistamines are the most frequently prescribed drugs for relieving AR symptoms [6] and are recommended as first-line therapy, alone or in combination with intranasal steroids [7-9]. Patient’s satisfaction is a key for successful management of allergic rhinitis improving compliance and clinical outcomes in a virtuous cycle [10,11]. Patients’ expectations may go, however, beyond symptomatic relief. Improving patient’s satisfaction implies a better understanding of the patient’s needs and a more accurate assessment of treatment benefit from the patient’s point of view. Patients’ needs may, for example, include in-depth information on the disease and its treatments as suggested by a study conducted in patients with intermittent AR that found that one out of five patients felt that they were poorly informed about the disease [6]. Regarding benefits, going beyond measurable efficacy to patient-relevant benefit is a new paradigm. Generally speaking, patients want to see a reduction of morbidity, improvement of quality of life, a decrease in symptoms and a decrease in treatment burden.

Recently, a new tool for the assessment of patient-defined benefit in the treatment of allergic rhinitis has been proposed and validated in English (AR-PBI for Allergic Rhinitis-Patient Benefit Index) [12]. The questionnaire consists of 25 non-redundant and non-overlapping items assessing patient-relevant needs and benefits and is suited for clinical practice and research. Patients with AR show a large spectrum of needs regarding treatment. AR-PBI is significantly correlated with treatment satisfaction, patient’s quality of life and treatment burden meaning that the more satisfied and the less burdened the patients were, the higher the benefit was. The AR-PBI has proven to be easy to understand.

The aim of the study was to validate the French language version of the AR-PBI and to assess the treatment-related expectations and benefits provided in patients with allergic rhinitis treated with H1-antihistamines in a real-life study.

## Methods

### Patient benefit index

The tool for the assessment of the patient benefit in AR was initially adapted from a validated generic patient benefit assessment tool in dermatology [13]. Items of the generic tool were replaced by disease-specific items after compilation of an item pool generated by patient interviews and reduction of the item pool by an expert panel of physicians and patients. The final version includes 25 non-redundant and non-overlapping items exploring patient-relevant therapy needs and benefits. Patients are asked to answer each of these 25 items at the start (Patient Needs Questionnaire, PNQ) and at the end of treatment (Patient Benefit Questionnaire, PBQ) on a five-point Likert scale. Patient-relevant therapy needs are scaled from 0 ‘not important at all’ to 4 ‘very important’ with a response option ‘does not apply to me’. At end of treatment, patients are asked to rate the extent of benefit achieved with the treatment, scaled from 0 ‘treatment did not help at all’ to 4 ‘treatment helped very much’. The Patient Benefit Index (PBI) is a global score ranging from 0 to 4 computed by dividing each rating on a need item by the sum of all ratings in the PNQ, multiplying this fraction with the respective benefit rating in the PBQ and summing these products. A PBI ≥ 1 is considered as a threshold of ‘relevant benefit’ as stated in a study of patients with psoriasis [12,14]. A French version of the PBI was obtained after a translation-back translation process [15].

### Study design

A random sample of physicians was asked to participate in the observational, prospective, longitudinal study BENEFICA. Physicians who volunteered to take part in the study should ask the first 4 consecutive patients seen in a routine visit who meet the inclusion criteria to enter the study. Patients must be at least 18 years old and be suffering from AR either documented by any relevant allergen specific IgE and/or prick test within the previous 5 years or by presenting AR with a symptomatic rhinitis occurring in a limited number of places at well-defined times (a season, for example) or during specific circumstances (mowing the grass, for example). Patients should not have been treated with antihistamines during the 8 days prior to inclusion in the study and should be prescribed with antihistamines on the day of inclusion. Antihistamines were administered orally. Given the observational nature of the study, the physician had a free choice in which antihistamine to prescribe. Institution of concomitant treatments with intranasal steroids, nasal vasoconstrictors or antileukotrienes was not allowed. Patients could however continue these treatments if they were ongoing at entry. Patients undergoing allergen immunotherapy or who had taken any such treatment but discontinued it less than 5 years ago were not allowed to enter the study.

### Collected data

Collected data included demographics, life habits, comorbidities frequently associated with AR (namely asthma, sinusitis, serous otitis media, urticaria, atopic dermatitis, sinus or nasal polyposis, according to the physician’s knowledge). Symptoms including rhinorrhea, a blocked nose, sneezing, nasal pruritus, eye pruritus, red eye, eye stinging, watery eyes and pharyngeal discomfort were rated on a 4-level scale (0: no symptoms, 1: mild symptoms, 2: moderate symptoms, 3: severe symptoms). AR was classified according to ARIA with persistent rhinitis defined by symptoms occurring for at least 4 days a week over at least 4 consecutive weeks during the last year. The physicians gave details on the AR treatment history and on the treatment that was prescribed at the end of the entry visit. Patients were provided with two sets of self-assessment questionnaires and were instructed to complete the first one at home immediately after the visit and before any intake of the prescribed antihistamine. The second set of self-assessment questionnaires was to be completed at home 14 days later or earlier in the event of treatment premature discontinuation. Both sets of self-assessment questionnaires had to be returned separately using the prepaid envelopes to the data center. Each set included an evaluation of AR-related discomfort on a 10 cm visual analog scale (0: no discomfort, 10: extreme discomfort), the miniRQLQ (Rhinoconjunctivitis Quality of Life Questionnaire, a 14-item questionnaire exploring the negative impact on daily life caused by AR symptoms and their consequences [16]), and either the PNQ for the first set or the PBQ for the second set. The French versions of the PNQ and PBQ are provided in the supporting information (See Additional file 1: Tables S1 and S2). The somnolence item was individualized from the miniRQLQ with negative impact on daily life rated on a 6-item scale (from 0: no negative impact on daily life to 6: huge negative impact on daily life). Data collected by physicians and self-assessment questionnaires were numbered similarly to allow reconciliation before analysis.

### Ethical considerations

This study was conducted in compliance with the Good Clinical Practices protocol and Declaration of Helsinki principles. In accordance with French law, formal approval from an ethical committee is not required for observational studies. Patients gave oral consent to participate after being informed about the study protocol by a written information sheet. Oral consent was to be documented in the patient’s medical file. The French “Commission Nationale Informatique et Libertés” [Data Protection Commission] gave its approval for the study.

### Statistics

Internal consistency, construct validity and external consistency of the French version of the AR-PBI were assessed. Internal consistency was evaluated by the Cronbach α coefficient which is an estimate of the reliability of a psychometric test, measuring if all items of the test are measuring the same underlying concept. A value of 0.80 or more is generally considered as the proof of excellent consistency. Construct validity was explored by principal component analysis (Varimax rotation). Correlations with change in miniRQLQ and discomfort (Pearson’s correlation coefficients) explored external validity of the PBI questionnaire. Patient-related needs (PNQ) and benefits (PBQ) were described and the AR-PBI was calculated for each patient. An AR-PBI ≥ 1 was used as the threshold of “relevant benefit” [12,14]. Statistical significance of evolution of discomfort and quality of life was tested by a Student’s *t* test for paired samples.

The statistical analysis used SAS 9.3 software (SAS Institute, Cary, NC, USA). Results are expressed as mean ± SD or number and percentages.

## Results

### Patients

Three thousands and two hundred patients filled-in both sets of self-assessment questionnaires. 111 out of 3200 patients did not meet the inclusion criteria. Therefore the analysis was conducted on 3089 patients who have been recruited by 912 physicians (general practitioners 84.6%, allergists 9.4%, other specialists 6.0%). Characteristics of patients are displayed in Table 1. According to the ARIA classification, rhinitis was mild and intermittent in 1.9% of patients, moderate/severe and intermittent in 10.3%, mild and persistent in 7.1%, moderate/severe and persistent in 80.7%. Concomitant diseases were asthma in 18.3% of patients, sinusitis in 13.5%, serous otitis media in 1.4%, atopic dermatitis in 11.9%, and nasal or sinus polyps in 5.5%.

**Table 1:** **Patients’ characteristics**

	**N = 3089**
Age (years)	38.8 ± 13.7
Males	1481 (48.2%)
Active smokers	642 (20.8%)
Passive smokers	507 (16.4%)
Occupational exposure to allergens	416 (14.1%)
Duration of allergic rhinitis (years)	14.5 ± 11.0
Perennial rhinorrhea	668 (22.3%)
ARIA classification of rhinitis	
Mild and intermittent	58 (1.9%)
Moderate and intermittent	314 (10.3%)
Mild and persistent	216 (7.1%)
Moderate and persistent	2463 (80.7%)
Concomitant diseases	
Rhinosinusitis	418 (13.5%)
Sero-mucous otitis	43 (1.4%)
Nose or sinus polyps	170 (5.5%)
ENT disease*	588 (19.0%)
Asthma	565 (18.3%)
Atopic dermatitis	366 (11.9%)

Results are expressed as mean ± SD for continuous variables and as a number (percentage) for categorical variables; *ENT disease includes rhinosinusitis, serous otitis media, and nose or sinus polyps.

### Symptoms

**Figure 1:**
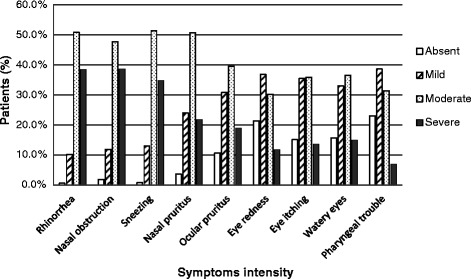
**Intensity of symptoms at entry in the study (n = 3089).** The percentage of patients is reported on the vertical axis. Symptoms are reported on the horizontal axis, from left to right: rhinorrhea, a blocked nose, sneezing, nasal pruritus, red eye, eye stinging, watery eye, pharyngeal discomfort. Symptom intensity (absent, mild, moderate, severe) is represented by columns: white columns correspond to absent; hatched columns correspond to mild; dotted columns correspond to moderate; and black columns correspond to severe.

At entry almost all patients presented rhinorrhea, sneezing and nasal obstruction (respectively 99.4%, 99.2% and 98.2%). Nasal pruritus was present in 96.4% of patients, eye pruritus in 89.4%, eye stinging in 84.9%, and watery eyes in 84.4%. Eye redness and pharyngeal discomfort were slightly less frequent (78.7% and 77.0% respectively). Figure 1 displays the distribution of symptoms intensity at entry. Patients reported a discomfort level of 7.2 ± 1.4 with a maximum of 10 and miniRQLQ was 46 ± 15 on a scale ranging from 0 to 84 showing an impairment of quality of life. Patients exhibited a mild degree of somnolence at entry (2.6 ± 1.6 on a scale ranging from 0 to 6), higher in patients with persistent than in those with intermittent rhinitis and more elevated in patients with severe symptoms than in patients with moderate symptoms.

### Treatments prescribed at entry visit

All patients were prescribed with oral antihistamines (99% bilastine), associated with a concomitant prescription of glucocorticoids in 15.2% and cromones in 9.2%). Self-reported compliance with antihistamine treatment was excellent with 96.3% of patients stating that they had taken the treatment regularly over the 14-day period of the study.

### Evolution of discomfort and quality of life

Quality of life significantly improved over the study period whereas discomfort decreased (Table 2). The mean discomfort level fell from 7.2 ± 1.4 to 3.1 ± 1.5 (p < 0.0001) and miniRQLQ decreased from 46 ± 15 to 15 ± 12 (p < 0.0001). All dimensions improved: activities, practical problems, nasal symptoms, eye disorders and other problems (p < 0.0001 for each dimension). The somnolence score increased in 113 patients (3.7%). In these patients, negative impact on daily life due to somnolence was moderate in 23 patients (score 3/6), fair in 17 patients (score 4/6), significant in 20 patients (score 5/6) and very significant in 5 patients (score 6/6).

**Table 2:** **Evolution of rhinitis over a 14-day course of antihistamines**

	**N = 3089**	
	**Baseline**	**End of study**
Discomfort (cm)	7.2 ± 1.4	3.1 ± 1.5*
MiniRQLQ	46.3 ± 14.5	14.7 ± 11.6*
Activities	11.0 ± 3.5	3.9 ± 3.0*
Practical problems	8.0 ± 2.2	2.7 ± 2.1*
Nasal symptoms	12.3 ± 3.3	4.0 ± 3.0*
Ocular symptoms	8.7 ± 4.7	2.4 ± 2.8*
Other problems	6.3 ± 4.3	1.8 ± 2.5*
Somnolence score	2.6 ± 1.6	0.8 ± 1.1*

Results are expressed as mean ± SD for continuous variables and frequency (percentage in documented data) for categorical variables. Discomfort is self-evaluated by the patient on a 10-cm visual analog scale; *p < 0.0001 between baseline and end of study.

### Results from the AR- PBI

#### Acceptability

The AR-PBI elicited a good response from patients which was adequate enough to produce significant data, since the percentage of missing data never exceeded 1% for any statement on the PNQ. As expected, there were differences in the ratings that the patients attributed to each statement; every patient had differing views on which statements were important or unimportant. Depending on the statement, the rate of “not concerned” patients varied from 0.3% to 36.4%. The statement yielding the highest percentage of “not concerned” responses was 36.4% for “To feel less depressed”.

#### Patient’s needs

Patients’ expectations are detailed in Table 3. Among afflicted patients (i.e. patients who did not select the response “not concerned” to the statement), there was a strong need to be healed (98.7%), not to have a runny nose (97.8%), and to be able to breathe more freely via the nose (97.1%). Relief from sneezing, nose stinging, burning or watery eyes were also expectations which received high mean ratings from the patients. Feeling less depressed, resuming a normal sexual life, spending less time on daily treatment, being less dependent on Doctors and clinic visits, or having fewer out-of-pocket treatment expenses received lower mean ratings from the patients.

**Table 3:** **Patients’ needs (PNQ before treatment initiation)**

	**m ± SD**	**Percentage of afflicted patients**	Percentage of quite/very important responses*
To be healed for all symptoms	3.5 ± 0.7	99.7%	98.7%
To no longer have a runny or blocked nose	3.4 ± 0.8	99.4%	97.8%
To be able to breathe through my nose more freely	3.3 ± 0.8	98.8%	97.1%
To not have sneezing impulses	3.1 ± 0.9	97.3%	93.3%
To not experience eye, nose or palate stinging anymore	3.1 ± 1.0	96.0%	92.3%
To be able to stay outdoors without symptoms	3.0 ± 0.9	96.8%	94.1%
To be able to engage in normal leisure activities	3.0 ± 1.0	94.9%	92.3%
To experience more enjoyment of life	2.9 ± 1.0	92.0%	89.8%
To have a treatment which is easy to use	2.9 ± 1.1	97.1%	89.2%
To have confidence in the therapy	2.9 ± 1.1	94.5%	89.1%
To not have burning or watery eyes anymore	2.9 ± 1.1	91.5%	87.8%
To be able to sleep better	2.8 ± 1.1	89.8%	84.6%
To be able to concentrate better at work	2.6 ± 1.1	80.1%	81.7%
To feel less fatigued or groggy	2.6 ± 1.2	89.6%	80.1%
To be more productive in everyday life	2.4 ± 1.2	82.2%	77.2%
To have fewer side effects	2.4 ± 1.2	79.5%	74.5%
To feel less burdened in your relationship	2.4 ± 1.2	82.8%	74.1%
To feel more comfortable in public	2.3 ± 1.2	83.9%	73.1%
To have no fear that the disease will become worse	2.3 ± 1.3	79.4%	72.8%
To be able to have a normal sex life	2.2 ± 1.3	66.0%	67.8%
To feel less depressed	2.2 ± 1.3	63.3%	66.9%
To feel less irritated	2.1 ± 1.2	76.8%	67.0%
To be less dependent on Doctor and clinic visits	2.1 ± 1.3	81.4%	65.0%
To spend less time on daily treatment	2.0 ± 1.3	79.8%	60.9%
To have fewer out-of-pocket treatment expenses	1.9 ± 1.4	74.2%	56.3%

Analysis of the Patient Needs Questionnaires (PNQ) filled in before treatment. Patients’ needs are ordered by decreasing importance. Needs are scaled from 0 ‘not important at all’ to 4 ‘very important’; *percentage calculated in afflicted patients.

#### Patient-related benefits

**Figure 2:**
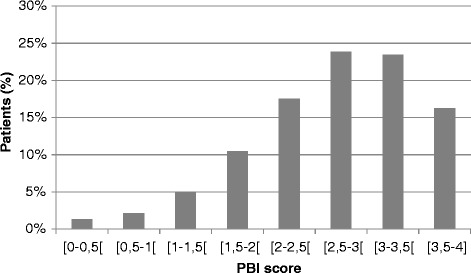
**AR-PBI distribution in patients with AR after a 14-day treatment period with antihistamines (n = 3089).** The percentage of patients is reported on the vertical axis while the patient benefit index (PBI) score is reported on the horizontal axis. This score ranges from 0 to 4 and is divided into 8 intervals.

AR-PBI was calculated in 3063 out of 3089 patients. They achieved a mean AR-PBI of 2.7 ± 0.8 (median 2.8) ranging from 0 to 4 (Figure 2). Applying a threshold of AR-PBI ≥ 1, 2956 patients (96.5%) received relevant benefit from antihistaminic treatment. Details on patient-related benefit are provided in Table 4. Maximal benefits were obtained for symptoms. There was no impact of tobacco consumption (p = 0.73), occupational allergen exposure (p = 0.38) on AR-PBI, nor of concomitant ENT disease (p = 0.85).

**Table 4:** **Patients’ benefits related to current antihistamine treatment**

	mean ± SD*	Percentage of patients helped rather/a lot**	Weighted subscore m ± SD***
To be healed for all symptoms	3.1 ± 0.9	92.8%	0.21 ± 0.11
To no longer have a runny or blocked nose	3.0 ± 0.9	91.9%	0.19 ± 0.11
To be able to breathe through my nose more freely	3.0 ± 1.0	91.6%	0.19 ± 0.11
To not have sneezing impulses	2.9 ± 1.0	89.9%	0.17 ± 0.10
To not experience eye, nose or palate stinging anymore	2.8 ± 1.0	88.0%	0.16 ± 0.10
To be able to stay outdoors without symptoms	2.9 ± 1.0	90.0%	0.16 ± 0.10
To be able to engage in normal leisure activities	2.8 ± 1.1	85.3%	0.15 ± 0.08
To have a treatment which is easy to use	2.7 ± 1.2	81.2%	0.14 ± 0.10
To experience more enjoyment of life	2.6 ± 1.2	81.8%	0.13 ± 0.08
To not have burning or watery eyes anymore	2.8 ± 1.1	85.0%	0.15 ± 0.09
To have confidence in the therapy	2.6 ± 1.2	80.5%	0.13 ± 0.09
To be able to sleep better	2.5 ± 1.3	76.0%	0.12 ± 0.09
To feel less fatigued or groggy	2.4 ± 1.2	75.6%	0.11 ± 0.07
To be able to concentrate better at work	2.4 ± 1.2	74.1%	0.10 ± 0.07
To be more productive in everyday life	2.4 ± 1.2	75.7%	0.10 ± 0.06
To feel less burdened in your relationship	2.3 ± 1.3	72.1%	0.09 ± 0.07
To have fewer side effects	2.2 ± 1.3	69.3%	0.09 ± 0.07
To have no fear that the disease will become worse	2.2 ± 1.3	69.2%	0.09 ± 0.07
To feel more comfortable in public	2.3 ± 1.3	72.2%	0.09 ± 0.07
To be able to have a normal sex life	2.0 ± 1.4	59.5%	0.08 ± 0.07
To feel less depressed	2.0 ± 1.3	62.0%	0.07 ± 0.06
To feel less irritated	2.1 ± 1.3	63.8%	0.07 ± 0.06
To be less dependent on Doctor and clinic visits	2.0 ± 1.3	63.2%	0.07 ± 0.06
To spend less time on daily treatment	2.0 ± 1.4	60.1%	0.07 ± 0.06
To have fewer out-of-pocket treatment expenses	1.8 ± 1.4	54.9%	0.06 ± 0.06
Global weighted PBI	2.7 ± 0.8		
Patients with global PBI ≥1	2956 (96.5%)		

Analysis of the Patient Benefits Questionnaires filled-in after antihistamine treatment. Patient’s benefits are ordered by decreasing importance of the corresponding need. Treatment-related benefits are scaled from 0 ‘did not help at all’ to 4 ‘helped a lot’. *Data are described as mean ± SD. **Percentage of patients with the need achieved by treatment from ‘rather helped’ to ‘helped a lot’ among afflicted patients at treatment initiation; ***PBI subscores weighted by the relative amount of the corresponding patient’s need.

#### Construct validity and internal consistency

Principal component analysis showed that three factors (social, physical and emotional) explained 67% of the global PNQ variance (See Additional file 2: Table S3). These factors did not all carry the same weight. The social factor explained 51%, the physical factor explained 12% and the emotional factor explained 4% of the global variance. Internal consistency was excellent (Cronbach alpha 0.96) showing that all questions were measuring the same underlying concept. Questions were not redundant, as shown by the distribution of Pearson’s correlation coefficients (See Additional file 3: Figure S1).

#### Concurrent validity

The AR-PBI was moderately correlated with changes in discomfort and quality of life over the 14-day treatment period (Pearson’s correlation coefficient −0.38 for discomfort and −0.45 for miniRQLQ, p < 0.0001 for both) suggesting that the PBI had a larger conceptual scope and measured more than symptoms and quality of life improvements. Change in discomfort and quality of life explained 21.3% of the AR-PBI global variance in a multiple regression model. The population was divided in quintiles according to PBI (Table 5). This showed that the higher the discomfort and the more impaired the quality of life at baseline, the higher the PBI would be (p < 0.0001). Moreover, patients with greater improvement in discomfort and mini-RQLQ had a higher PBI.

**Table 5:** **Discomfort and mini-RQLQ at baseline and changes from baseline to end of study according to Patient Benefit Index**

	**Q1**	**Q2**	**Q3**	**Q4**	**Q5**	**p**
	**N = 615**	**N = 602**	**N = 619**	**N = 613**	**N = 614**	
Global PBI	1.4 ± 0.5	2.3 ± 0.2	2.8 ± 0.1	3.1 ± 0.1	3.7 ± 0.2	-
	[0.00-2.01]	[2.01-2.57]	[2.57-2.983]	[2.983-3.384]	[3.384-4.00]	
Discomfort at baseline	6.7 ± 1.6	7.0 ± 1.4	7.2 ± 1.4	7.4 ± 1.3	7.5 ± 1.4	<0.0001
Mini RQLQ at baseline	40.3 ± 14.4	43.0 ± 13.3	46.8 ± 13.4	50.1 ± 14.1	51.5 ± 14.2	<0.0001
Change in discomfort at baseline compared to end of study	−2.9 ± 1.9	−3.7 ± 1.7	−4.2 ± 1.6	−4.5 ± 1.8	−5.0 ± 1.9	<0.0001
Change in miniRQLQ at baseline compared to end of study	−20.9 ± 15.5	−26.6 ± 13.0	−32.4 ± 13.6	−36.1 ± 15.2	−42.0 ± 15.4	<0.0001
Worsening in somnolence having a negative impact on daily life	55 (9.1%)	24 (4.0%)	14 (2.3%)	13 (2.1%)	7 (1.2%)	<0.0001

The population was divided in quintiles (Q1 to Q5) according to PBI. PBI extent and mean value is described in each quintile. Changes in discomfort and quality of life are calculated as change in 14-day value minus baseline so that improvements are expressed as negative values. Results are expressed as mean ± SD or number (%). Comparisons used ANOVA for continuous variables or chi^2^ for categorical variables.

#### AR-PBI in known sub-groups

Patients willing to pursue antihistamines after the 14-day course (N = 2537) had a higher PBI score than the 94 patients willing to discontinue (2.7 ± 0.8 versus 1.4 ± 0.9, p < 0.0001). Patients reporting a worsening in negative impact on daily life due to somnolence (N = 113) had a significantly lower PBI (p < 0.0001) associated with poorer outcome and poorer compliance.

## Discussion

This study evaluated the properties of the French version of AR-PBI, highlighted that patient-related benefit comprises more than solely symptomatic relief and underlined the relationship between treatment-related benefit and quality of life. AR-PBI was moderately correlated with improvement of quality of life or decrease in discomfort, therefore complementing and not competing with the two outcomes. In addition, this study provided new insights on short-term patient-related benefits in patients receiving antihistamine agents. The French AR-PBI has proven to be well understood and easy to use by patients, as demonstrated by the low number of missing responses.

Our study demonstrated that the French version of AR-PBI has the same properties as the index English version [12] despite methodological differences between the validation methods. The index validation study was cross-sectional with patients receiving various treatments and some others being burden-free at the time of the study. Conversely, our study was prospective and longitudinal with all patients complaining of AR symptoms and none being receiving antihistamines when entering the study. All were prescribed with an antihistamine for 14 days.

Patients’ characteristics were similar between the two studies, except for the sex ratio with 62.5% of women in the index study. 87.8% of our patients suffered from persistent rhinitis, a number higher than in the DREAMS (74%) [17] and INSTANT studies [2] (50%). The INSTANT study aimed at estimating the AR prevalence in the general population whereas the DREAMS study was conducted in patients visiting ENT or allergy specialists for AR as in the BENEFICA study. The DREAMS study recruited 76% of patients with moderate to severe AR, a number significantly lower than in our population (91%). Again the difference could be due to our inclusion criteria which required antihistamines to be started at inclusion visit. We found a 18.3% prevalence of asthma, a number slightly lower than in other studies [2,3,18,19] but quite similar to the DREAMS study (24%) [16]. Quality of life was impaired in our study as already found in many studies using various tools (SF36 [20], SF-12 [2], RQLQ [4,20] or specific questionnaires exploring discomfort, consequences for sleep, eating, mood, daily activities, occupation and leisure activities [6]). In addition sleep disorders and somnolence have already been described in AR patients [2,21-23]. In a French cohort, the prevalence of sleep complaints was one of 5 in mild AR, 1 out of 2 in moderate-severe AR (and 18% in a control group). The prevalence of sleep disorders (insomnia) was around 14% in mild AR and 40-42% in moderate-severe AR (and 16% in a control group) [22].

The spectrum of patient’s needs was very large and extended beyond symptomatic relief. Correlations between PBI and change in miniRQLQ or discomfort were significant but moderate. Principal component analysis provided some keys for a better understanding of patient’s needs. Indeed, the main dimension that explained 51% of the global variance was related to social items such as feeling less depressed, to be able to better concentrate at work, to have no fear that the disease would become worse, to be more productive, to feel more comfortable in public, to feel less burdened in a relationship and to be able to have a normal sex life. Clearly these expectations extended further than solely symptomatic relief and thus were not perceived by other tools including miniRQLQ. In addition an “emotional” factor including items such as feeling less fatigued or groggy, being able to sleep better, experiencing more enjoyment of life and being able to engage in normal leisure activities are not perceived to a sufficient extent by other tools and must not be neglected.

Antihistamines proved to be very effective in relieving symptoms and improving quality of life. Beyond these well-known properties, antihistamines have been demonstrated to produce a very substantial benefit from the patient’s perspective since almost all patients reported a clinically relevant benefit defined by an AR-PBI ≥ 1. Neither anchor-based methods nor analyses based on PBI distribution allow us to discuss this threshold which has been proposed by the PBI authors [12]. Anchor-based methods compare patients according to a clinical outcome (improvement versus no change or deterioration for example). Given that symptoms and discomfort dramatically decreased over the study period in most patients, such methods were not applicable. Applying methods based on PBI distribution (such as half of standard deviation) [24], we found a value of 0.4 which was even lower than the proposed threshold. Antihistamines have exceeded the patient’s expectations for improvement beyond symptomatic relief.

Our study has some limitations. Firstly, the population of AR patients was not randomly selected but was constituted from patients visiting a participating Doctor during the survey. Secondly physicians were not selected at random but volunteered for the study. These limitations however are unlikely to have influenced the results of the study because patient needs and patient-defined benefit were not evaluated by the physician but by the patient him/herself. Patients’ symptomatic profiles included the classic triad of rhinorrhea, sneezing and nasal obstruction but the frequency of these symptoms was higher than in other studies conducted in France which reported a prevalence of 85% for these symptoms versus 99% in our study. This could be due to the inclusion criteria, which boosted selection of patients who were more symptomatic and who were actively seeking effective treatment. Another limitation is the short duration of the follow-up which was limited to 15 days. We cannot assume that these short-term benefits would continue to exist if the follow-up was extended. The study was not randomized and in real life observational conditions, it was not possible to have a control group. Its first aim, however, was not to evaluate the benefits provided by anti-histamines but to provide additional understanding of the way that patients perceive these benefits and specifically to explore dimensions other than symptomatic relief. This study may help clinicians to understand why medication may fail despite good symptomatic relief or why they may succeed even if symptom relief is only modest. The lack of a control group does not weaken these conclusions. Finally self-assessment questionnaires were administered at home and we cannot exclude the notion that some retrospective completion may have occurred.

## Conclusion

The French version of the AR-PBI has been validated. AR-PBI allows the patient to estimate his own needs and to assess the benefit of treatment in terms of his own expectations. This original point of view may complete the usual tools assessing treatment efficacy including quality of life questionnaires and clinical scores.

## Additional files

Additional file 1: Table S1. French version of the PBI - Patient Needs Questionnaire (PNQ). French version of the PBI – Patients Needs Questionnaire (PNQ). **Table S2.** French version of the PBI - Patient Benefit Questionnaire (PBQ). French version of the PBI – Patients Benefit Questionnaire (PBQ). Additional file 2: Table S3. Principal component analysis of the patient’s needs in allergic rhinitis. Principal components analysis of the patient’s needs (N = 3089). Patient’s needs are related to 3 factors: Factor 1- Social; Factor 2- Physical; Factor 3- Emotional. Items with higher correlation coefficient to each factor are in bold. Additional file 3: Figure S1. Distribution of Pearson’s correlation coefficients between PNQ items. Distribution of Pearson’s correlation coefficients for the questions of the Patient Needs Questionnaire (PNQ).

## Abbreviations

AR: Allergic rhinitis
ARIA: Allergic rhinits and its impact on asthma
PBI: Patient benefit index
PBQ: Patient benefit questionnaire
PNQ: Patient needs questionnaire
RQLQ: Rhinoconjunctivitis quality of life questionnaire
