# Supplementary material for: Evaluation of patients’ expectations and benefits in the treatment of allergic rhinitis with a new tool: the patient benefit index – the benefica study
Source: Allergy Asthma Clin Immunol. 2015 Feb 26;11(1):8. doi: 10.1186/s13223-015-0073-1 (PMC4349226; doi:10.1186/s13223-015-0073-1)
Supplement: Additional file 1: Table S1. — French version of the PBI - Patient Needs Questionnaire (PNQ). French version of the PBI – Patients Needs Questionnaire (PNQ). Table S2. French version of the PBI - Patient Benefit Questionnaire (PBQ). French version of the PBI – Patients Benefit Questionnaire (PBQ). [file 13223_2015_73_MOESM1_ESM.docx]

## Table S1 - French version of the PBI - Patient NeedS Questionnaire (PNQ)

*Qu’attendez-vous d’un traitement pour la rhinite allergique ?*

*Si vous n’êtes pas concerné par la question (par exemple si vous n’avez pas les yeux qui pleurent), cochez la case « Non concerné ».*

|  | Non concerné | Pas important | Peu important | Assez important | Important | Très important |
| --- | --- | --- | --- | --- | --- | --- |
| Etre soulagé de l’ensemble des symptômes | □ | □ | □ | □ | □ | □ |
| Ne plus avoir le nez bouché ou le nez qui coule | □ | □ | □ | □ | □ | □ |
| Respirer plus facilement par le nez | □ | □ | □ | □ | □ | □ |
| Ne plus avoir de crises d’éternuements | □ | □ | □ | □ | □ | □ |
| Avoir un traitement facile à prendre | □ | □ | □ | □ | □ | □ |
| Pouvoir rester en plein air | □ | □ | □ | □ | □ | □ |
| Ne plus avoir les yeux, le nez ou le palais qui démangent | □ | □ | □ | □ | □ | □ |
| Avoir des activités de loisir normales | □ | □ | □ | □ | □ | □ |
| Faire confiance au traitement | □ | □ | □ | □ | □ | □ |
| Mieux profiter de la vie | □ | □ | □ | □ | □ | □ |
| Ne plus avoir les yeux qui pleurent / qui brûlent | □ | □ | □ | □ | □ | □ |
| Mieux dormir | □ | □ | □ | □ | □ | □ |
| Se sentir moins fatigué / moins faible | □ | □ | □ | □ | □ | □ |
| Se sentir plus présentable aux yeux des autres | □ | □ | □ | □ | □ | □ |
| Etre moins perturbé dans sa relation avec les autres | □ | □ | □ | □ | □ | □ |
| Etre plus productif dans la vie de tous les jours | □ | □ | □ | □ | □ | □ |
| Diminuer la fréquence des visites chez le médecin | □ | □ | □ | □ | □ | □ |
| Etre capable de mieux se concentrer au travail | □ | □ | □ | □ | □ | □ |
| Perdre moins de temps à prendre son traitement chaque jour | □ | □ | □ | □ | □ | □ |
| Avoir moins d’effets indésirables qu’avec les traitements précédents | □ | □ | □ | □ | □ | □ |
| Ne pas avoir peur que la maladie s’aggrave | □ | □ | □ | □ | □ | □ |
| Se sentir moins irritable | □ | □ | □ | □ | □ | □ |
| Diminuer les dépenses de soin | □ | □ | □ | □ | □ | □ |
| Mener une vie sexuelle normale | □ | □ | □ | □ | □ | □ |
| Se sentir moins déprimé | □ | □ | □ | □ | □ | □ |

## Table S2 - French version of the PBI - Patient Benefit Questionnaire (PBQ)

*Sur les différents points listés ci-dessous, quelle aide vous a apporté le traitement que vous avez pris au cours des 2 dernières semaines ?*

|  | Aucune aide | Aide peu importante | Aide assez importante | Aide importante | Aide très importante |
| --- | --- | --- | --- | --- | --- |
| Etre soulagé de l’ensemble des symptômes | □ | □ | □ | □ | □ |
| Ne plus avoir le nez bouché ou le nez qui coule | □ | □ | □ | □ | □ |
| Respirer plus facilement par le nez | □ | □ | □ | □ | □ |
| Ne plus avoir de crises d’éternuements | □ | □ | □ | □ | □ |
| Avoir un traitement facile à prendre | □ | □ | □ | □ | □ |
| Pouvoir rester en plein air | □ | □ | □ | □ | □ |
| Ne plus avoir les yeux, le nez ou le palais qui démangent | □ | □ | □ | □ | □ |
| Avoir des activités de loisir normales | □ | □ | □ | □ | □ |
| Faire confiance au traitement | □ | □ | □ | □ | □ |
| Mieux profiter de la vie | □ | □ | □ | □ | □ |
| Ne plus avoir les yeux qui pleurent / qui brûlent | □ | □ | □ | □ | □ |
| Mieux dormir | □ | □ | □ | □ | □ |
| Se sentir moins fatigué / moins faible | □ | □ | □ | □ | □ |
| Se sentir plus présentable aux yeux des autres | □ | □ | □ | □ | □ |
| Etre moins perturbé dans sa relation avec les autres | □ | □ | □ | □ | □ |
| Etre plus productif dans la vie de tous les jours | □ | □ | □ | □ | □ |
| Diminuer la fréquence des visites chez le médecin | □ | □ | □ | □ | □ |
| Etre capable de mieux se concentrer au travail | □ | □ | □ | □ | □ |
| Perdre moins de temps à prendre son traitement chaque jour | □ | □ | □ | □ | □ |
| Avoir moins d’effets indésirables qu’avec les traitements précédents | □ | □ | □ | □ | □ |
| Ne pas avoir peur que la maladie s’aggrave | □ | □ | □ | □ | □ |
| Se sentir moins irritable | □ | □ | □ | □ | □ |
| Diminuer les dépenses de soin | □ | □ | □ | □ | □ |
| Mener une vie sexuelle normale | □ | □ | □ | □ | □ |
| Se sentir moins déprimé | □ | □ | □ | □ | □ |
