# Supplementary material for: Evaluation of patients’ expectations and benefits in the treatment of allergic rhinitis with a new tool: the patient benefit index – the benefica study
Source: Allergy Asthma Clin Immunol. 2015 Feb 26;11(1):8. doi: 10.1186/s13223-015-0073-1 (PMC4349226; doi:10.1186/s13223-015-0073-1)
Supplement: Additional file 2: Table S3. — Principal component analysis of the patient’s needs in allergic rhinitis. Principal components analysis of the patient’s needs (N = 3089). Patient’s needs are related to 3 factors: Factor 1- Social; Factor 2- Physical; Factor 3- Emotional. Items with higher correlation coefficient to each factor are in bold. [file 13223_2015_73_MOESM2_ESM.docx]

**Table S3 - Principal components analysis of the patient’s needs in allergic rhinitis**

Principal components analysis of the patient’s needs (N=3089). Patient’s needs are related to 3 factors: Factor 1- Social; Factor 2- Physical; Factor 3- Emotional. Items with higher correlation coefficient to each factor are in bold.

|  | Factor 1 | Factor 2 | Factor 3 |
| --- | --- | --- | --- |
| To be able to stay outdoors without symptoms | 0.18 | **0.75** | 0.19 |
| To no longer have a runny or a stuffed-up nose | 0.04 | **0.79** | 0.31 |
| To not have itching on the eyes, nose or palate anymore | 0.22 | **0.82** | 0.04 |
| To not have burning or watery eyes anymore | 0.28 | **0.74** | 0.02 |
| To be healed for all symptoms | 0 | **0.74** | 0.33 |
| To be able to breathe through my nose more freely | 0.10 | **0.64** | 0.45 |
| To feel less fatigued or groggy | 0.48 | 0.33 | **0.68** |
| To have confidence in the therapy | 0.33 | 0.27 | **0.65** |
| To have an easily applicable treatment | 0.32 | 0.21 | **0.67** |
| To be able to sleep better | 0.39 | 0.28 | **0.65** |
| To experience a greater enjoyment of life | 0.41 | 0.40 | **0.58** |
| To be able to engage in normal leisure activities | 0.37 | 0.48 | **0.50** |
| To feel less depressed | **0.72** | 0.13 | 0.38 |
| To be able to concentrate better at work | **0.67** | 0.28 | 0.38 |
| To not have sneezing impulses | 0.34 | **0.57** | 0.29 |
| To have no fear that the disease will become worse | **0.67** | 0.17 | 0.40 |
| To be more productive in everyday life | **0.71** | 0.27 | 0.37 |
| To be less dependent on doctor and clinic visits | **0.76** | 0.11 | 0.28 |
| To have fewer side effects | **0.72** | 0.15 | 0.29 |
| To feel less irritated | **0.80** | 0.16 | 0.31 |
| To have fewer out-of-pocket treatment expenses | **0.83** | 0.05 | 0.18 |
| To need less time for daily treatment | **0.85** | 0.08 | 0.16 |
| To feel more comfortable showing yourself | **0.79** | 0.28 | 0.10 |
| To be less burdened in your partnership | **0.80** | 0.30 | 0.10 |
| To be able to have a normal sex life | **0.78** | 0.17 | 0.18 |
| **Dimension** | Social | **Physical** | **Emotional** |
| Eigenvalue | 12.67 | 2.96 | 1.06 |
| Proportion of variance explained (%) | 50.67 | 11.86 | 4.24 |
