# Supplementary material for: Evaluation of patients’ expectations and benefits in the treatment of allergic rhinitis with a new tool: the patient benefit index – the benefica study
Source: Allergy Asthma Clin Immunol. 2015 Feb 26;11(1):8. doi: 10.1186/s13223-015-0073-1 (PMC4349226; doi:10.1186/s13223-015-0073-1)
Supplement: Additional file 3: Figure S1. — Distribution of Pearson’s correlation coefficients between PNQ items. Distribution of Pearson’s correlation coefficients for the questions of the Patient Needs Questionnaire (PNQ). [file 13223_2015_73_MOESM3_ESM.docx]

**Figure S1 - Distribution of Pearson’s correlation coefficients between PNQ items**

Distribution of Pearson’s correlation coefficients for the questions of the Patient Needs Questionnaire (PNQ).
